# Editorial: Cancer Informatics Toward Precision Medicine

**DOI:** 10.3389/fmed.2020.576611

**Published:** 2020-11-25

**Authors:** Fan Zhang, Xiaogang Wu, Wencong Chen, Youping Deng

**Affiliations:** ^1^Department of Family Medicine & Institute for Translational Research, University of North Texas Health Science Center, Fort Worth, TX, United States; ^2^University of Texas Monroe Dunaway Anderson Cancer Center, Houston, TX, United States; ^3^Department of Biostatistics, Vanderbilt University Medical Center, Nashville, TN, United States; ^4^Department of Quantitative Health Sciences, University of Hawaii, Honolulu, HI, United States

**Keywords:** cancer informatics, precision medicine, biomarker discovery, system biology, data mining

This Research Topic focuses on cancer informatics and is intended to present and discuss innovative reports, methodologies, tools, and algorithms that enable precision cancer medicine related to genomics, proteomics, and metabolomics (“omics”) data being generated on clinical tumors. These omics data in combination with approaches, tools and algorithms have the potential to transform cancer care through high-throughput analysis of patient-derived tumors and promote “precision” medicine through tumor molecular profiling.

The first article of this Topic (Schwartz et al.) introduces inteGREAT, an algorithm to integrate transcript and protein abundance data and detect differential biomarkers between multiple cancer subtypes. Integration of information by high-throughput measurements of transcripts or proteins is useful for discovery of transcript- or protein-based prognostic and diagnostic biomarkers but challenging because previous studies have shown low correlation between the transcript and protein levels. Therefore, it is critical to effectively combine information gathered by the genome-scale and the protein-level measurements for cancer biomark discovery. inteGREAT is a generic algorithm to measure inter-network similarity and report differential information. For example, the authors in this study used inteGREAT for biomarker detection from transcriptome and proteome data in different cancer subtypes. The authors also showed robustness and accuracy of inteGREAT using simulations controlling for multiple sources of biological noise. With multiple analyses, the authors demonstrated that integration of transcriptome and protein interactomes can improve reliability of biomarker discovery. The flexibility of inteGREAT enables analysis of networks from various biological sources, including the epigenome, CNVs, and mutation data etc. This algorithm is a powerful tool to further cancer biomarker discovery and may help doctors fully deliver on the promise of precision oncology and aid in therapeutics advancements.

Biomarker panels has been widely used as biomarker identification for cancer. In Fang, Zhu, Hu et al., Plasma MicroRNA Pair Panels were used as novel biomarkers for detection of early stage breast cancer. The authors performed next-generation sequencing miRNA expression profiling on three pooling samples of plasma from breast cancer, benign lesion, and normal. They first identified and validated 13 microRNAs using real-time quantitative reverse transcription-polymerase chain reaction (RT-qPCR) in a cohort of 53 breast cancer, 40 benign lesions, and 38 normal cases. And then used the pairwise miRNA ratios as biomarkers to classify breast cancer. Lastly they selected panels of miRNA ratios combined with age which could yield high performance for comparison between breast cancer and normal/non-cancer groups. The results show that MicroRNA pairs identification is an effective method for biomarker discovery in early stage breast cancer, especially when distinguishing cancer from benign lesions.

The third article of this Topic (Fang, Zhu, Khadka et al.) introduced usage of serum biomarkers for non-small Cell Lung Cancer (NSCLC) diagnosis. The expression levels of prolactin (PRL), CEA, and CYFR21 in serum were assayed by ELISA across the blood samples from 44 NSCLC cases and 44 healthy controls. The authors first measured the performance for usage of single marker. Among the three serum biomarkers, CEA displayed the highest AUC, followed by PRL and CYFRA21. And then they used a logistic regression to explore the prediction power fir combination of the two or three serum biomarkers. They found that combining all the three markers yielded a best diagnostic performance for cancer patients. These statistics and machine learning methods such as logistic regression and receiver operating characteristic (ROC) analyses can also be applied to biomarker discovery for other cancers.

The last article of this Topic (Iuliano et al.) used system biology method to obtain better insights into breast cancer biological mechanisms. System biology method is an approach in biomedical research to understanding the larger picture by putting entire biological systems together—such as protein complexes, metabolic pathways, or gene regulatory networks. In this article, the authors presented novel screening-network methods that predict patient survival outcome by screening key survival-related genes and tested the capability of the approaches using METABRIC dataset. They first identified a subset of genes by using variable screening techniques on gene expression data. Then, they performed Cox regression analysis by incorporating network information associated with the selected subset of genes. They also demonstrated that combining different types of screenings and integrating an additional omic layer, such as copy number aberrations could further improve prediction performance. They found that the approaches could use only few potential biomarkers to discriminate patients in high-and low-risk groups and help provide more precise prognoses for clinicians.

We hope that the reader will find this Research Topic a useful reference for data mining, machine learning, biomarker discovery, and network analysis for precision oncology.

## Author Contributions

FZ, XW, WC, and YD contributed to the design and implementation of the Research Topics. FZ wrote the paper with input from all authors. All authors contributed to the article and approved the submitted version.

## Conflict of Interest

The authors declare that the research was conducted in the absence of any commercial or financial relationships that could be construed as a potential conflict of interest.

